# Antifungal Activities of Different Essential Oils and Their Electrospun Nanofibers against *Aspergillus* and *Penicillium* Species Isolated from Bread

**DOI:** 10.1021/acsomega.2c05105

**Published:** 2022-10-14

**Authors:** Dilara Devecioglu, Mustafa Turker, Funda Karbancioglu-Guler

**Affiliations:** † Faculty of Chemical and Metallurgical Engineering, Department of Food Engineering, Istanbul Technical University, 34449 Maslak, Istanbul, Turkey; ‡ Pak Group, R & D Center, Köseköy Mahallesi, Ankara Cad. No. 277, 41310 Kartepe, Kocaeli, Turkey

## Abstract

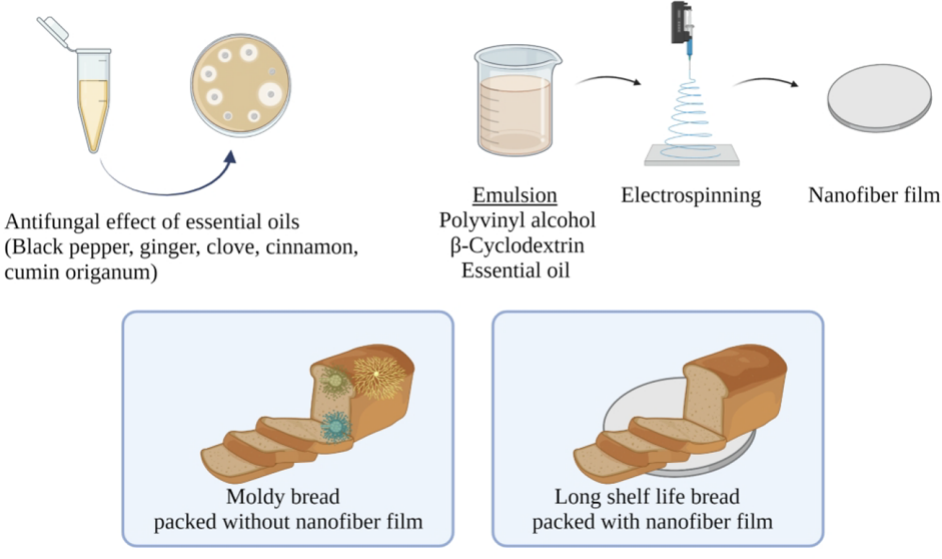

Mold growth, especially *Aspergillus* spp. and *Penicillium* spp., deteriorates the quality of bakery products. Essential oils (EOs) have been categorized as good natural antimicrobials. Hereby, this study aimed to evaluate the antifungal activity of six EOs, ginger, cumin, cinnamon, black pepper, origanum, and clove, and their volatile compounds against fungal strains isolated from bread: *Penicillium carneum* DDS4, *Aspergillus flavus* DDS6, and *Aspergillus niger* DDS7 by disc diffusion and disc volatilization methods, respectively. Among EOs, cumin, cinnamon, origanum, and clove were found to be effective against fungal strains, and their minimum inhibitory concentration (MIC) and minimum fungicidal concentration (MFC) were determined. The observed lowest MIC value of EOs was obtained at 1000 μg/mL concentration, and the lowest MFC value was obtained from the results of clove at a concentration of 1000 μg/mL. Based on the MIC and MFC values, clove and cinnamon EOs were found to be more effective at lower concentrations. Electrospun nanofiber films of clove and cinnamon were produced with 6% poly(vinyl alcohol) (PVA), 2% β-cyclodextrin (β-CD), and 2% EO to overcome the unfavorable sensory impact of EOs on food products. The inhibitory activity of cinnamon EO film (2.64–2.51 log(CFU/mg)) was considerably lower than clove EO film (3.18–3.24 log(CFU/mg)) against *P. carneum* DDS4 and *A. niger* DDS7. Furthermore, these nanofiber films prevented fungal growth on bread samples visibly and were shown to be an alternative application for active food packaging.

## 1 Introduction

Food safety is a common public health issue due to the increase in food-borne diseases. Approximately 600 million people fall ill from consuming contaminated food, and 90% of recorded cases result from only diarrheal disease, causing 230,000 deaths every year.^1^ In recent years, there has been an observable effort to increase food products’ quality and safety by using natural antimicrobials by virtue of their inhibition ability of bacterial and fungal growth.^2,3^ Plants, animals, bacteria, algae, and fungi are the main sources of natural antimicrobials, and the antimicrobial effectiveness of plant-based products, including EOs, was shown in many studies.^4−7^ EOs are secondary metabolites of plants, and the presence of several components in their composition has been demonstrated as the main reason for their antimicrobial activity.^8,9^ In addition to their antimicrobial effects, EOs may also exhibit different activities such as antiviral,^10,11^ antimycotic,^12^ antiparasitic,^13,14^ and insecticidal.^15,16^ However, these activities, which are mainly dependent on the composition affected by various factors, including extraction method,^17,18^ harvesting time,^19,20^ geographical origin, and plant parts,^21,22^ differ even for the same plant.

The antimicrobial activity of EOs has been reported in several studies.^23−26^ Although most of the studies have focused on the antibacterial activities of EOs, their antifungal activity has been a subject of recent studies.^27−30^ Although there are studies showing the *in vivo* antifungal effect of EOs for several foods,^31,32^ it is also necessary to focus on studies showing the antifungal effect of EOs without changing the food composition or direct EO treatment. There are some generally mentioned mechanisms of action, including the destruction of the cell wall, damage to protein and membrane, changes in permeability, and inhibition of synthesis pathways.^33−35^

Bread is one of the most consumed products in the bakery industry. However, it has a short shelf life, and some physicochemical changes occur during storage. As a result, bread quality deteriorates gradually, and that loss is associated with microbial spoilage in general.^36^*Penicillium* spp. and *Aspergillus* spp. are the most common spoilage molds for bread.^37,38^ Therefore, recent technologies have been getting attention to inhibit spoilage and increase shelf life without any quality loss.

Food packaging is an important part of the food industry both to ensure safety and to reduce food waste.^39,40^ EOs are classified as generally recognized as safe, and they have been incorporated into packaging material as antimicrobial agent.^41^ However, their usage is often limited due to their strong flavors, and above a certain dosage they may not be acceptable organoleptically. Nowadays, new approaches provide admissible solutions for the utilization of EOs as food packaging components. Therefore, encapsulation of EOs is important to mask the undesirable flavor of EOs, to distribute EOs in the food matrix, and increase both the solubility and antimicrobial activity.^42−45^ In addition, the fact that EO encapsulation is stated as a green solution in terms of food safety has led researchers to focus on this issue.^46^ Cyclodextrins are among the agents used for encapsulation,^47^ and β-CD is a widely used natural CD type due to its cavity size, cost, and ability to encapsulate hydrophobic compounds.^42,48^ However, a limited number of studies demonstrated the antifungal activity of encapsulated EOs.^49−51^

On the other hand, the release and effect mechanism of EOs may change according to the type of packaging.^52^ Nanoencapsulation of antimicrobial agents with polymers is one of the proposed methods for encapsulation. The used polymers can be synthetic or biobased like lipids, polysaccharides, and protein. The edible and nontoxic properties of biobased polymers are their major advantages, and they also provide the ability to control shelf life. PVA is a nontoxic and biocompatible polymer and is commonly selected for electrospinning solution preparation of antimicrobial agents.^53,54^ Many strategies have been developed to produce nanomaterial, and electrospinning is a well-referenced and attractive method.^55^ The electrospun nanofibers provide several advantages, including a large surface-to-volume ratio, high porosity, and favorable structure characteristics.^56,57^

In this respect, the aim of this study is to investigate and compare the antifungal activity of essential oils and their electrospun nanofibers against molds isolated from bread: *Penicillium carneum, Aspergillus flavus*, and *Aspergillus niger*. Hence, this study differentiates from other studies by using isolated and identified target microorganisms, examining the antifungal activity, and encapsulating essential oils using β-CD. Additionally, it is thought that this study will increase the understanding of active packaging by applying produced electrospun nanofibers to bread.

## 2 Results and Discussion

### 2.1 Identification of Fungal Cultures Isolated from Bread

A total of 12 different fungal cultures were isolated from selected bread samples. After the microscopic observation, five selected fungal cultures were molecularly identified (Table 1).

**Table 1:** Identification of Fungal Cultures Isolated from Bread

culture	species	Gen Bank code	identities (%)
DDS1	*Penicillium* spp.	AJ005677	568/569 (99)
DDS4	*Penicillium carneum*	KC009821	565/567 (99)
DDS6	*Aspergillus flavus*	JX232269	565/568 (99)
DDS7	*Aspergillus niger*	EU440778	584/588 (99)
DDS12	*Aspergillus* spp.	GU988901	513/513 (100)

Among them, three fungal cultures showing a homology of 99% to *Penicillium carneum* DDS4, *Aspergillus flavus* DDS6, and *Aspergillus niger* DDS7 were selected for further experiments. They also were mentioned as the most common bread spoilage fungi in refs (37) and (38).

### 2.2 Antifungal Properties of Essential Oils

#### 2.2.1 Disc Diffusion and Disc Volatilization Method

The assessment of the antifungal activity of the EOs was carried out by both diffusion and vapor assays. As shown in Table 2, except ginger EO (GEO), all remaining EOs exhibited antifungal activity. While origanum EO (OEO) exhibited greater inhibition zones against all tested fungal isolates, black pepper EO (BPEO) showed slight inhibition against only *P. carneum* DDS4. However, Li et al.^58^ determined the notable antifungal activity of black and white pepper; it can be interpreted that this result may be due to the type of tested fungal culture and compositional differences of EOs. Figure S1 shows images of disc diffusion plates conducted to determine the antifungal activity of EOs.

**Table 2:** Inhibition Zones (Diameter, cm) of Essential Oils (EOs), and Volatile Components of Essential Oils (VEO) (Mean ± SD)

	*Penicillium carneum* DDS4				*Aspergillus flavus* DDS6				*Aspergillus niger* DDS7			
	EO		VEO		EO		VEO		EO		VEO	
EOs§	3rd day	6th day	3rd day	6th day	3rd day	6th day	3rd day	6th day	3rd day	6th day	3rd day	6th day
**BPEO**	4.7 ± 0.3^c^	NE^e^	2.3 ± 0.3^c^	1.6 ± 0.6^c^	NE^e^,*	NE^d^	NE^d^	NE^c^	0.9 ± 0.1^d^	0.8 ± 0.14^d^	NE^c^,#	NE^c^
**CEO**	6.8 ± 0.1^b^	7.0 ± 0.4^b^	4.2 ± 1.0^b^	3.0 ± 0.4^b^	3.9 ± 0.1^c^	3.9 ± 0.1^b^	2.2 ± 0.5^c^	1.7 ± 0.3^b^	3.8 ± 0.4^c^	2.7 ± 0.2^c^	1.6 ± 0.3^b^	0.7 ± 0.2^b^
**CLEO**	7.3 ± 0.1^b^	7.3 ± 0.1^b^	3.9 ± 0.0^b^	3.0 ± 0.6^b^	4.3 ± 0.5^c^	4.6 ± 0.6^b^	2.5 ± 0.0^c^	2.0 ± 0.2^b^	4.1 ± 0.1^c^	3.8 ± 0.1^b^	2.1 ± 0.0^b^	0.9 ± 0.0^b^
**CUEO**	>9^a^,#	3.7 ± 1.06^c^	>9^a^	>9^a^	>9^a^^3^	1.6 ± 0.7^c^	>9^a^	NE^c^	6.6 ± 0.3^b^	1.0 ± 0.3^d^	NE^c^	NE^c^
**GEO**	1.7 ± 0.5^d^	1.2 ± 0.3^d^	NE^d^	NE^d^	NE^d^	NE^d^	NE^d^	NE^c^	NE^d^	NE^e^	NE^c^	NE^c^
**OEO**	>9^a^	>9^a^	>9^a^	>9^a^	7.5 ± 0.4^b^	>9^a^	5.3 ± 0.6^b^	4.7 ± 0.6^a^	>9^a^	>9^a^	6.3 ± 0.6^a^	6.2 ± 0.8^a^

* NE: Noninhibitory effect.

# >9: Strong inhibition effect. No growth has been detected.

a–e Values within the same column with different superscript small letters are significantly different (*p* < 0.05).

§ BPEO: black pepper essential oil, CEO: cinnamon essential oil, CLEO: clove essential oil, CUEO: cumin essential oil, GEO: ginger essential oil, OEO: origanum essential oil.

Moreover, the results indicated that fungal cultures showed varied sensitivity to EOs. In the meantime, the sensitivity of a culture to different EOs demonstrated variation as parallel to the study of Davari and Ezazi.^59^ Here, *P. carneum* DDS4 was found to be less resistant against EOs among studied fungal cultures, while remarkable growth of *A. flavus* DDS6 and *A. niger* DDS7 were stated under the same conditions. Until now, few studies have compared the susceptibility of strains to EOs. While it was found that there were susceptibility differences between *Penicillium* spp.,^60,61^ the difference between various genera should be found to be acceptable.

Generally, the increases in the incubation period decreased the inhibitory effect of EOs in both diffusion and vapor assays. As can be seen from Table 2, it was possible to conclude that OEO had a prolonged inhibitory effect among four EOs.

On the other hand, the disc volatilization method provided knowledge about the antifungal ability of volatile components in the composition of EOs due to the absence of direct contact with fungal culture. This is an important feature to provide biocontrol with the help EOs. A study by Perumal et al.^62^ showed that EO vapor had a non-negligible effect on inoculated mango, and its effectiveness increased with increasing concentration, as expected. Though volatile components possessed antifungal activity, it was seen that EOs in the liquid form exhibited stronger antifungal activity than their vapor phase. The study carried out to investigate the antibacterial activity of EOs against several pathogens also found that the volatile component showed a remarkable but usually lower antibacterial effect than the liquid form.^63^ It is known that EOs show their antimicrobial effect through various mechanisms.^64^ The less effective volatile components may be due to the difference in their mechanism of action.

In light of these findings, cinnamon EO (CEO), clove EO (CLEO), cumin EO (CUEO), and OEO were chosen for further study.

#### 2.2.2 Poisoned Culture Method

After the initial screening of the antifungal activity of EOs, the MIC and MFC values of selected EOs were determined. As seen in Table 3, the MIC and MFC values of EOs ranged from 1000 to 3000 μg/mL. Except for CUEO, the MFC values of the remaining EOs were generally higher than their MIC value. In addition, the MFC values of CEO were higher than the MIC values against all tested fungal strains.

**Table 3:** Minimum Inhibitory Concentration (MIC) and Minimum Fungicidal Concentration (MFC) Values of Four Essential Oils (EOs) for Identified Fungal Cultures

	MIC (μg/mL)			MFC (μg/mL)		
EOs^b^	*P. carneum*^a^ DDS4	*A. flavus*^a^ DDS6	*A. niger*^a^ DDS7	*P. carneum*^a^ DDS4	*A. flavus*^a^ DDS6	*A. niger*^a^ DDS7
CEO	1000	1500	1000	1500	2000	1500
CLEO	1000	1000	1500	1000	1500	1500
CUEO	3000	2000	1500	3000	2000	1500
OEO	2000	2000	1500	3000	3000	1500

a *P. carneum, Penicillium carneum; A. flavus, Aspergillus flavus; A. niger, Aspergillus niger*.

b CEO: cinnamon essential oil, CLEO: clove essential oil, CUEO: cumin essential oil, OEO: origanum essential oil.

CLEO exhibited high antifungal activity with a MIC value of 1000 μg/mL against *P. carneum* DDS4 and *A. flavus* DDS6, and its highest MIC value was 1500 μg/mL for *A. niger* DDS7. Pinto et al.^65^ reported MIC values of clove EO for *Aspergillus* spp. between 0.32 and 0.64 μL/mL, and MFC values were determined as 1.25 μL/mL. Purkait et al.^66^ determined the MFC values of clove and cinnamon EOs against *Aspergillus niger*. Compared to the current study, the MFC values of EOs were found to be lower (65–73 μg/mL). This observed difference may be related the use of different *Aspergillus* species and differences in EO composition. Similar to this situation, Melo et al.^67^ found that the MIC value of *Ocimum gratissimum* L. EO against *S. aureus* changed based on the its strains (1000 μg/mL for ATCC 6538 and 2000 μg/mL for 5B). Another study performed by 12 different strains of *E. coli* showed that the MIC value of the same EO changed between 50 and 250 ppm.^68^ Raybaudi-Massilia et al.^69^ also concluded that different MIC/MBC values of EOs are available in the literature, and not only the specific strain but also the method applied were effective. They also commented that these parameters make it difficult to compare with the literature.

When the MIC and MFC values of the citrus-derived EOs were analyzed against *Penicillium* spp. and *Aspergillus* spp., these values were found to be the same, or the MFC value was higher. The MIC and MFC values determined for *Aspergillus* species were found to be in the range of 560–1130 and 560–2250 μg/mL, respectively, and it has been observed that there are similarities with the results of some EOs in the current study.^70^ OEO exhibited the same MIC (2000 μg/mL) and MFC (3000 μg/mL) values for *P. carneum* DDS4 and *A. flavus* DDS6. Manso et al.^71^ reported the MIC values of cinnamon and origanum EOs against *A. flavus* as 100 and 800 ppm, respectively, which were lower than the current study. On the other hand, Vitoratos et al.^61^ determined MIC value of origanum as 0.02 μL/mL (20 μg/mL for density: 1 g/mL) for *Botrytis cinerea*, Kocić-Tanackov et al.^72^ found 2 μL/mL (2000 μg/mL for density: 1 g/mL) for *Aspergillus versicolor.* These different results seen in the literature may be due to the difference in EO composition. For example, while carvone was the major compound (18.05%) of origanum in the study mentioned above,^72^ the isomerized form of carvone, carvacrol (57.22%), was found to be noticeably different in the composition of used OEO in the current study.

In another study, the MIC values of cinnamon and clove EOs for *Aspergillus parasiticus* were found to be 1000 and 1500 ppm, respectively, and were similar to the obtained findings. However, the MIC value for the EOs of cumin, black pepper, and ginger could not be determined in the studied concentration range of 500–2500 ppm.^27^ Apart from the differences that may arise from the composition of EOs, it has been interpreted that the reason for the detection of high MIC values by *in vitro* studies may be the interaction of phenolic components with the food matrix.

Although OEO and CUEO were found to be highly effective against fungal strains by the disc diffusion method, MIC and MFC values showed that the required concentration of OEO and CUEO to inhibit fungal cultures were higher than CEO and CLEO. Similar situations have been reported in the literature. In the study examining the antibacterial effect of *Lippia grandis* EO, although the *Escherichia coli* ATCC 35218 inhibition zone (29.3 mm) was higher than that for *Enterococcus faecalis* ATCC 29212 (13 mm), the MIC value for *E. faecalis* (0.57 mg/mL) was found to be lower than that for *E. coli* (1.15 mg/mL).^73^ In addition, Melo et al.^67^ reported that EOs with high inhibition zones in their study gave a high MIC value result.

### 2.3 Nanofiber Film Characterization

It was found that PVA, the polymer used in this study, formed a higher-quality fiber, and the parameters were optimized^74^ as in the publication of Wen et al.^42^ The nanofiber films were developed by incorporating PVA (6%), EO (2%), and β-CD (1%). From the operation conditions, the voltage was adjusted to 14–16 kV, the distance was 12 cm, and the flow rate was 0.4 mL/h. In this study, the EO concentration was determined on the basis of studies about nanofiber production with the most optimum properties. While Seydim and Sarikus^75^ could see no antimicrobial effect with 1% EO, the study of Wen et al.^42^ used a cinnamon EO concentration in the range of 1–3% and decided to use 2% for an antimicrobial effect. PVA and β-CD concentrations were also determined as the most optimum according to the viscosity and conductivity of the electrospinning solution.

**Figure 1:**
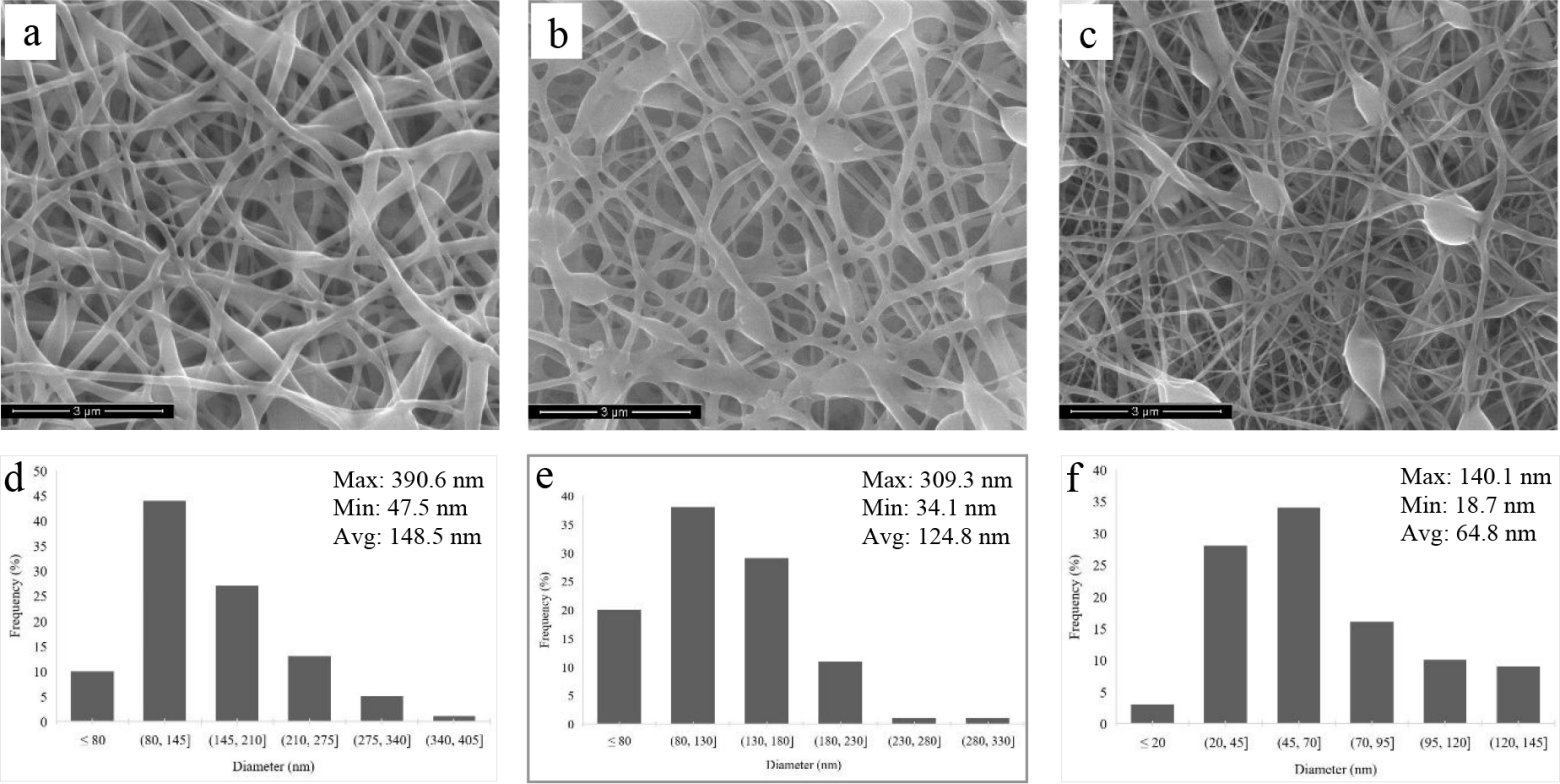
SEM images of (a) poly(vinyl alcohol) (PVA), (b) PVA/cinnamon essential/β-cyclodextrin (β-CD), and (c) PVA/clove essential oil/β-CD nanofibers.

The SEM images of nanofiber films of PVA, CEO, and CLEO are represented in Figure 1. After the incorporation of β-CD and EO with PVA, some bead formation was presented, unlike PVA nanofiber film (Figure 1a). The possible reason for this situation was expressed as a relationship between polymer concentration and the viscosity of the polymer solution.^76^ Munhuweyi et al.^77^ also observed beads on the nanofibers’ SEM images, including β-CD, PVA, chitosan, and EO. The diameter distributions of all produced nanofibers are presented in Figure 1d–f. As seen here, the average diameter of the PVA nanofiber (148.5 nm) was close to the average diameter of PVA/CEO/β-CD nanofiber (124.8 nm). However, when the diameters of 100 different fibers belonging to the PVA/CLEO/β-CD nanofiber were examined, the average diameter was observed to be half of the average diameter of PVA and PVA/CEO/β-CD nanofibers. In the study of Tavassoli-Kafrani et al.,^78^ while the average fiber diameter of the gelatin nanofiber film obtained by electrospinning was 83.5 nm, they concluded that the fiber diameter slightly increased by incorporation of the EO into nanofiber contrary to the current study. As Lin et al.^53^ indicated, the characteristic differences of the electrospinning solution may be the reason for this result.

**Figure 2:**
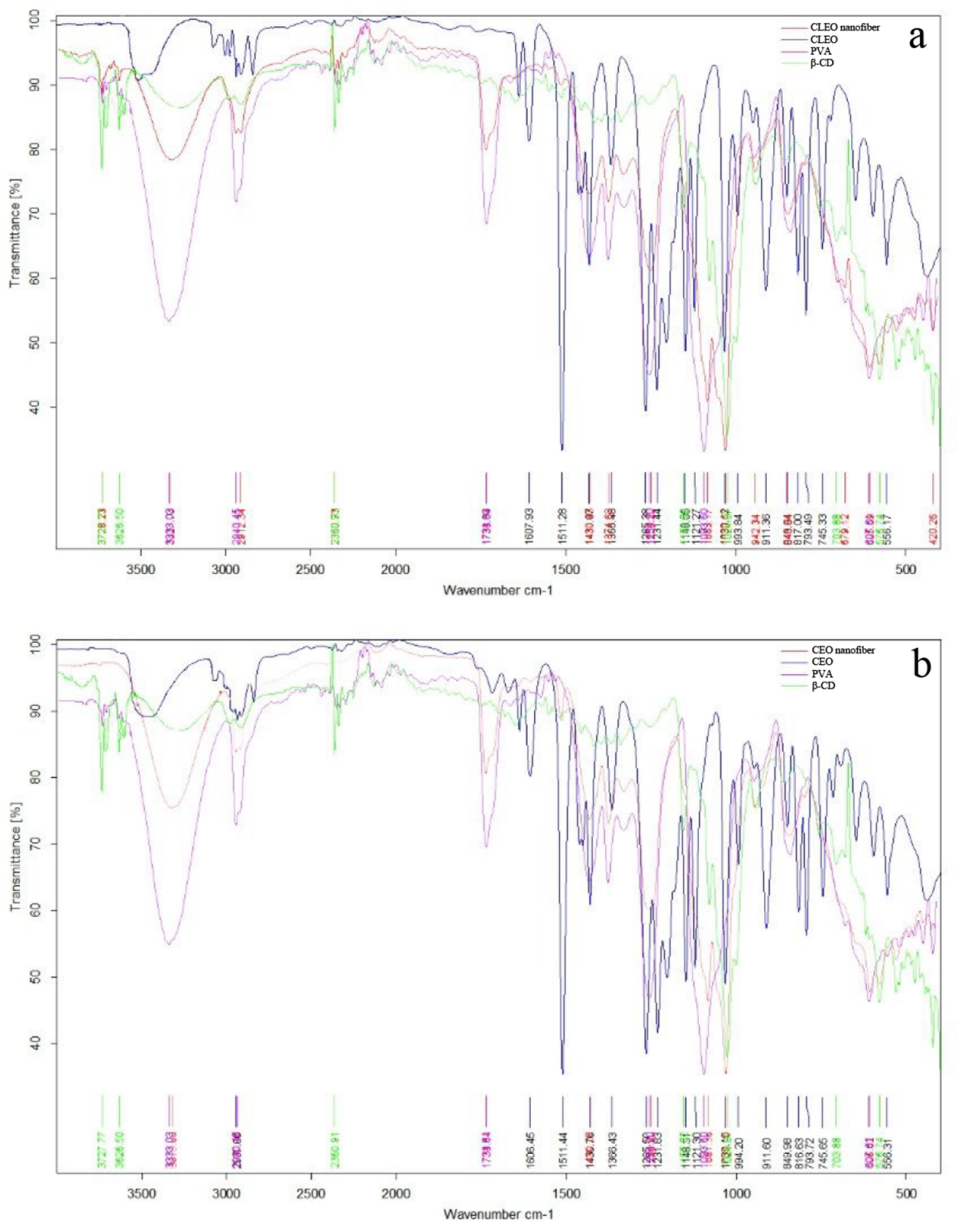
FTIR spectra of poly(vinyl alcohol), β-cyclodextrin, (a) clove essential oil (CLEO) nanofiber and CLEO, and (b) cinnamon essential oil (CEO) nanofiber and CEO.

The FTIR spectra of EO nanofiber film and its components, PVA, β-CD, and EO, were interlaced (Figure 2). It is seen that the majority of characteristic absorption peaks appeared between 1700 and 500 cm^–1^ and around 3300 cm^–1^. As the control group of the current study, PVA nanofiber film (purple line), bands were observed at 3333 and 2940 cm^–1^ representing O–H and C–H bonds,^79^ respectively. The study of Wen et al.^42^ also showed peaks at almost the same points. In additon, the important bands at 1424 and 1085 cm^–1^ were correlated with bands seen at 1432 and 1093 cm^–1^, representing the stretching of C–H and C–O in the C–O–H bond, respectively.

The study of Pan et al.^80^ with the same results of PVA spectra and Wen et al.^42^ detected specific bands of β-CD between 1030 and 1080 cm^–1^ (coupling stretching of C–O and C–C) and at 3300–3500 cm^–1^ (stretching of C–H) in the analogy of the current study.

As shown in Figure 2a,b, the spectra of the EO nanofiber film exhibited some characteristics of the absorption bands from EO, PVA, and β-CD. The band seen in CEO nanofiber film spectra at 1030 cm^–1^ was specific to β-CD, which proved the presence of β-CD in the film composition (Figure 2b). On the other hand, the bands at 2930, 1430, and 600 cm^–1^ were highly specific to both film and CEO. Similar to the FTIR spectra of the CEO nanofiber film, common peaks were observed for the CLEO nanofiber film (Figure 2a). Most of the nanofiber film’s specific bands belong to either PVA or β-CD. The bands at 1430 cm^–1^ represented the C–H bond, and the bands at 942 cm^–1^ indicated the presence of an α-1,4 bond.

According to these two spectra, CEO and CLEO had similarities; however, some of the observable specific peaks of both CEO and CLEO disappeared on nanofiber film spectra. It was explained by Lin et al.^53^ and Wen et al.^42^ as a result of encapsulation with β-CD. Therefore, this situation demonstrates the sign of a successfully produced EO and β-CD complex.

### 2.4 Antifungal Activity of Nanofiber Films

According to the antifungal activity of the essential oils, the most resistant fungal culture, *A. niger* DDS7, was followed by *A. flavus* DDS6 and *P. carneum* DDS4. Therefore, the antifungal activity of produced CEO and CLEO nanofiber films was tested against the most resistant and most sensitive cultures, *A. niger* DDS7 and *P. carneum* DDS4. As seen in Table 4, none of the nanofiber films showed complete inhibition of tested fungi. CLEO nanofiber film was insufficient, contrary to CLEO nanofiber film against *A. niger* DDS7, and was similar to PVA nanofiber film. On the other hand, CLEO nanofiber film was found to be effective against *P. carneum* DDS4 as compared to control film PVA. CEO nanofiber film demonstrated 0.6 log and 1.06 log reduction in regard to PVA for *A. niger* DDS7 and *P. carneum* DDS4, respectively. Therefore, the observed colonies’ size was smaller than colonies counted for PVA and CLEO nanofiber film. Similar to results obtained by screening the antifungal activity of EO, *P. carneum* DDS4 was detected as the most sensitive fungal culture against EO nanofiber films. Differences in the results of antimicrobial effects were observed when the EO was integrated into the nanofiber film, as the case of CLEO nanofiber film. The most important parameter that enables EO-integrated nanofilms to show antimicrobial effect is the release of EO from the film. According to studies, it has been seen that the release ability changes based on the components in the EO composition.^81,82^ Although both cinnamon and clove EO contain high levels of eugenol, the difference in their minor constituents may have caused variation in their release ability and a change in the antimicrobial effect of their nanofiber films.

**Table 4:** Antifungal Activity of Nanofiber Films (log(CFU/mg)) against *A. niger* and *P. carneum*

nanofiber films^a^	*A. niger* DDS7	*P. carneum* DDS4
PVA	3.24	3.57
PVA/CLEO/β-CD	3.24	3.18
PVA/CEO/β-CD	2.64	2.51

a PVA: Poly(vinyl alcohol), β-CD: β-cyclodextrin, CLEO: clove essential oil, CEO: cinnamon essential oil.

It is seen in the literature that there are studies using different polymers or nanocomposite components to test their effect on food model as packaging material.^83^ Bodbodak et al.^84^ also produced nanofibers by electrospinning, using polylactic acid and hydroxypropyl methylcellulose as polymer, and examined its antibacterial activity by including pomegranate peel extract as an antimicrobial component. Its antifungal activity and application as food packaging are not included, but its effectiveness against *Staphylococcus aureus* and *Escherichia coli* has been demonstrated with the addition of extract. Besides the plant extracts, although there are a certain number of studies on nanofibers prepared with PVA and β-CD together with EO, similar to the current study, the majority of the studies were carried out to examine their antibacterial effect. In a study examining the antifungal activity, the nanofiber film composed of PVA, chitosan, β-CD, and essential oil showed 47–54% inhibition of *Botrytis* sp. Cinnamon EO nanofiber film had a higher antifungal effect than oregano, and because of this, it has been commented that cinnamon had better entrapment into the β-CD cavity. Therefore, EO components may release better, and their solubility may be improved.^77^ It has been interpreted that this may be one of the reasons for the antifungal difference observed between the clove and cinnamon nanofibers examined in this study. In order to observe the high antifungal effect of EO in nanofiber, the EO concentration may be increased, as seen in the study of Yilmaz et al.^85^ They showed the activity of chitosan nanoparticle samples loaded with EOs against *Alternaria alternata* and observed inhibition of mycelial growth. Although the effectiveness increased by increasing the concentration of loaded EO, the implication of higher EO concentration into nanofiber film should be unfeasible due to the affected nanofiber film and solutions’ characteristics.

While the usability of the produced films as food packaging is interpreted in the studies, the changes observed in packed food products are not seen in each study. Guo et al.^86^ have been investigated the effect of polylactic acid packaging films from an antibacterial point of view for meat products. On the other hand, Aman Mohammadi et al.^87^ indicated that zein, polylactic acid, and hydroxypropyl methylcellulose nanofibers incorporated with Zenian EO could be evaluated as a potential food packaging material by increased antibacterial activity against *Escherichia coli* and *Staphylococcus aureus* after addition of EO. Although several studies about food packaging materials with antimicrobial activity are available, antifungal packaging materials have received less attention.^88^ The *in vivo* antifungal effects of CLEO and CEO nanofiber films were tested with organic bread samples. The visible fungal growth on packed and nonpacked bread samples stored under sterile conditions at 25 °C was checked during a 6-day storage period. In addition to studies stating that EOs are considered as GRAS, there are also studies indicating that cinnamon and clove EOs are considered as GRAS.^89−91^ Besides, eugenol, the major component of the cinnamon (70.80%) and clove (81.93%) EOs used in this study, is accepted as GRAS^92,93^ and used as a food preservative in many countries and regions.^94^ As stated by Munetaka et al..^90^ having no toxic effects to mammals as a result of *in vivo* studies also makes these EOs available for use in food packaging.

**Figure 3:**
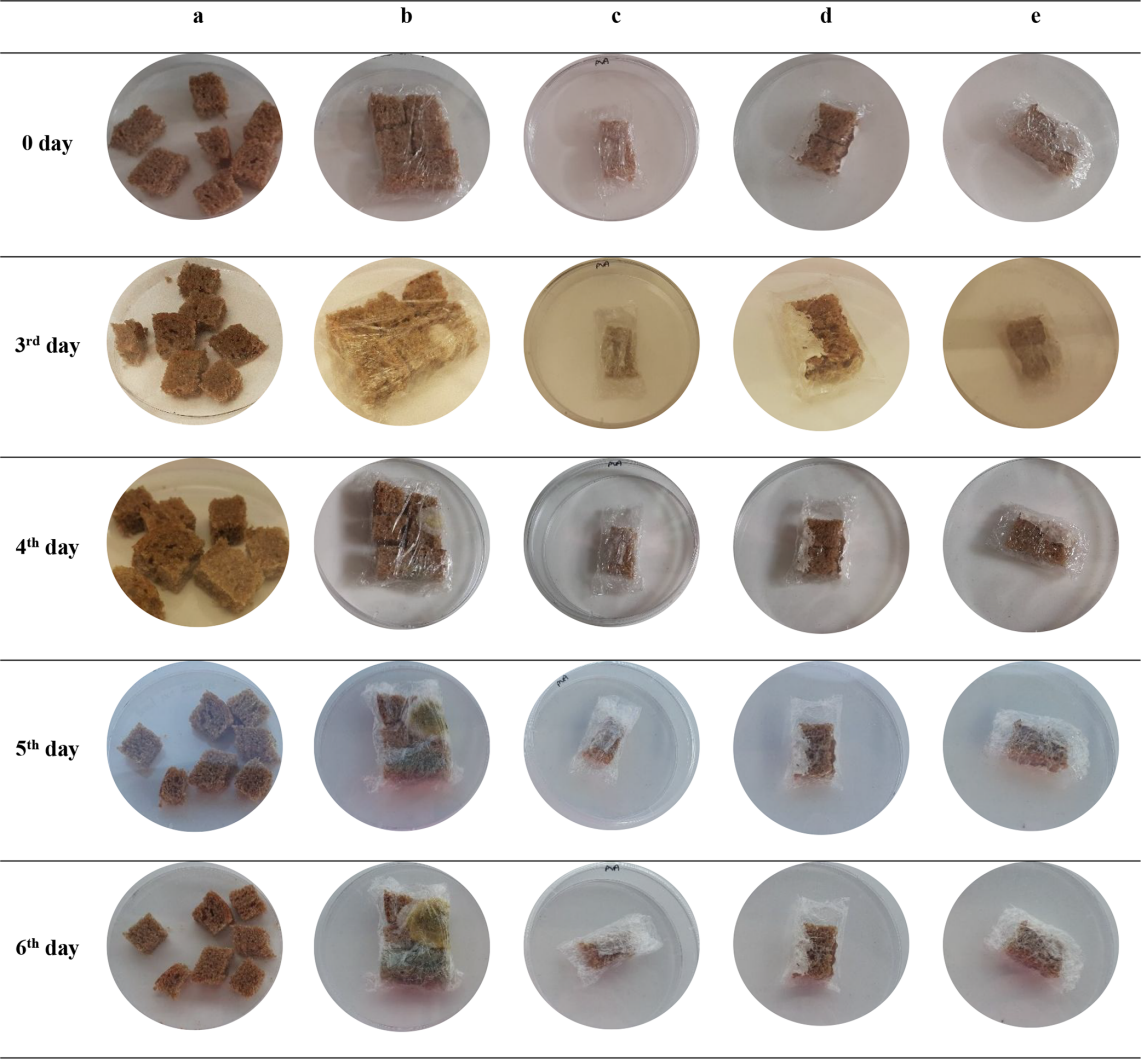
Appearance changes of different bread samples stored at 25 °C: (a) unpacked bread; (b) packed bread with cling wrap; (c) packed bread with poly(vinyl alcohol) (PVA) nanofiber film; (d) packed bread with PVA/clove essential oil/β-cyclodextrin (β-CD) nanofiber film; (e) packed bread with PVA/cinnamon essential oil/β-CD nanofiber film.

The fungal growth started on the third day of storage on bread packed with cling wrap, as seen in Figure 3, and the observed fungal growth increased day by day during storage. However, there was no fungal growth on bread without cling wrap due to loss of moisture, which is an important criterion for fungal growth. When the bread group packaged with cling wrap was examined, it was concluded that packaging with PVA also slowed down the fungal growth but it was not as effective as the film containing EO as the storage time increased. In other words, slices of bread packed with either CLEO or CEO nanofiber films showed no sign of any fungal growth.

In the study of Wen et al.,^42^ cinnamon EO nanofiber film, including PVA and β-CD as similar to the current study, was used for packaging of strawberries to check its efficiency on strawberries’ shelf life and quality. During the storage period at 4 °C, strawberries conserved their weight and firmness and besides shelf life was found to be prolonged contrary to samples either left unpacked or packed with fresh-keeping film. Both shelf life and sensory properties may be positively affected by the use of nanofiber films.

Overall, this phenomenon is an area still requiring development; therefore, research has been continuing. In spite of examining quality parameters during storage^80^ and antibacterial effecst,^53^ nanofiber film usage potential for the antifungal activity should be a concern of scientists as well, considering food security.

## 3 Conclusion

The antifungal activities of several EOs were tested against *P. carneum* DDS4, *A. flavus* DDS6, and *A. niger* DDS7. CLEO and CEO exhibited high inhibition efficiency among tested EOs. The PVA/CLEO/β-CD and PVA/CEO/β-CD nanofiber films were produced successfully, and it has been found that they prevent fungal growth during bread storage. The results of this study shed light on the use of nanofiber films for food packaging. It would provide the opportunity to develop packaging material consisting of EOs with antimicrobial activity as a bioactive agent. Besides, toxicity and sensory analysis tests should be done as a further study, and sustainability should be ensured with food products with extended shelf life.

## 4 Materials and Methods

### 4.1 Materials

Packaged bread products (organic, whole wheat, and sliced bread) close to the expiry date were collected from the local market. Black pepper essential oil (0.876 g/mL, containing 23.57% β-caryophyllene, 15.80% limonene D, 12.41% δ-3-carene+myrecene β, and 12.17% α-pinene), cinnamon leaf essential oil (1.027 g/mL, containing 70.80% eugenol), clove bud essential oil (1.004 g/mL, containing 81.93% eugenol and 12.28% β-caryophyllene), cumin seed essential oil (0.945 g/mL, containing 38.10% cumin aldehyde, 15.13% *p*-mentha-1,4-dien-7-al, and 12.03% *p*-cymene), ginger essential oil (0.815 g/mL, containing 30.55% zingiberene α and 18.84% α-curcumene + β-sesquiphellandrene), and origanum essential oil (0.944 g/mL, containing 57.22% carvacrol and 15.59% *p*-cymene) were kindly provided by Aromsa Besin Aroma ve Katkı Maddeleri Sanayi Ticaret A.Ş. (Kocaeli, Turkey). They were stored in the dark at 4 °C until analysis.

PVA (purity: 95.40%, 87.16% hydrolysis) was purchased from ZAG (Istanbul, Turkey), and β-CD (≥97%) was obtained from Sigma-Aldrich (Saint Louis, MO). Other chemicals used in the study were purchased from Merck (Darmstadt, Germany).

### 4.2 Isolation and Identification of Fungal Cultures from Bread

The isolation of fungal cultures from collected bread samples was performed by spreading the direct plating method using Dichloran Rose Bengal Chloramphenicol agar (Merck, Darmstadt, Germany) and Dichloran 18% Glycerol agar (Merck, Darmstadt, Germany). Purification was performed with malt extract agar, including 20 g/L glucose (Merck, Darmstadt, Germany). For their molecular identifications, DNA was extracted with Biospeedy Fungal DNA kit according to the instructions of the manufacturer. Molecular identification was carried out by amplifying the internal transcribed spacer region (ITS) using ITS1-5.8S rRNA and ITS2 (5′TCCTCCGCTTATTGATATGC3′) as forward and (5′GGAAGTAAAAGTCGTAACAAGG3′) as reverse primers for real-time polymerase chain reaction (QPCR). QPCR amplification was performed under the following conditions: initial denaturation at 95 °C for 10 min, 45 cycles at 95 °C for 15 s, 53 °C for 20 s, and final extension at 98 °C for 40 s. QPCR products were purified using the PCR Purification Kit following the manufacturer’s instructions. Sanger Dideoxy Sequence Termination Method using ABI Prism 377 DNA Sequencing Analyzer (Applied Biosystems, ABD) was used to analyze DNA sequences. Sequences for the 18S and ITS region were compared with the sequences available in National Center for Biotechnology Information (NCBI) using the online BLAST tool.^94^

### 4.3 Antifungal Properties of Essential Oils

#### 4.3.1 Disc Diffusion and Disc Volatilization Method

The antifungal activities of EOs against *Penicillium carneum* DDS4, *Aspergillus flavus* DDS6, and *Aspergillus niger* DDS7 were evaluated using disc diffusion and disc volatilization methods with a slight modification of Wen et al.^42^ Briefly, 100 μL of a prepared spore suspension (peptone water including 1 g/1 L Tween 80) containing approximately 10^5^ spores/mL was inoculated to Czapek dox agar (CZ) plates (Ø = 90 mm) individually. Inoculated plates were allowed to dry for 20 min at room temperature (CZ: 10 mL czapek concentrate, 1 g of dipotassium phosphate (K_2_HPO_4_), 30 g of sucrose, 17.5 g of agar in 1 L of distilled water, and czapek concentrate: 30 g of sodium nitrate (NaNO_3_), 5 g of potassium chloride (KCl), 5 g of magnesium sulfate heptahydrate (MgSO_4_·7H_2_O), 0.1 g of ferrous sulfate heptahydrate (FeSO_4_·7H_2_O), 0.1 g of zinc sulfate heptahydrate (ZnSO_4_·7H_2_O), and 0.05 g of copper sulfate pentahydrate (CuSO_4_·5H_2_O) in 100 mL of distilled water).

The sterilized filter disc (Ø = 6 mm) was placed on the inoculated CZ plate’s surface for the disc diffusion method and the inside surface of another plate’s medium-free cover for the disc volatilization method. Then, 5 μL of pure EO was added to the disc. These plates were sealed with parafilm to eliminate EO vapor leakage and incubated at 25 °C for 6 days. The diameter of the inhibition zone was measured after 3 days and 6 days of incubation.

#### 4.3.2 Poisoned Culture Method

The determination of essential oils’ antifungal activity was carried out by using the method described by Kocić-Tanackov et al.^72^ CZ 1% Tween 20 medium was cooled to 45 °C after sterilization, and CEO, CLEO, CUEO, and OEG in different concentrations (500, 750, 1000, 1500, 2000, 3000, and 5000 μg/mL) were added separately. After EO was dispersed in the medium, it was poured into a Petri plate (Ø = 60 mm). The plates, including medium without essential oil, were indicated as control. The sterilized filter paper disc (Ø = 6 mm) was placed at the center of the medium, and 1 μL of spore suspension, including 10^5^ spores/mL, was inoculated on a disc. The plates were sealed with parafilm and left for incubation at 25 °C for 14 days. The daily radial colony growth diameter was measured.^72^

MIC was determined as the minimum concentration that prevents fungal growth at the end of 14 days. Then the parafilm was removed from plates in which there was no colony growth, and they were left for a further 16 days of incubation. The filter paper discs without fungal growth after 30 days were transferred to a CZ medium free from EO, and they were incubated at 25 °C for 5 days to determine the MFC.

### 4.4 Electrospinning Process

The preparation of electrospinning solutions and performed processes was adapted from Wen et al.^42^ First, a 6% PVA solution was prepared by dissolving in distilled water under constant stirring by using a magnetic stirrer (IKA, RCT classic) at 700 rpm and 80 °C for 3 h. Then, 2% β-CD for CEO and CLEO was added and dissolved in PVA solution at 700 rpm and 55 °C for 1 h. After that, 2% EO was put inside the mixture, and the solution was stirred at 55 °C and 700 rpm for 2.5 h. As a control, a pure PVA solution was prepared.

The electrospinning procedure was driven with a 20-gauge steel needle at a distance of 12 cm, 0.4 mL/s flow rate, and 14–16 kV voltage. The nanofiber film was collected on a collecting plate covered by aluminum foil. The characterization of EO nanofilms was examined by Fourier transform infrared spectrophotometry (FTIR, Bruker Tensor II) in the range of scanning spectra from 500 to 4000 cm^–1^ and scanning electron microscopy (SEM, FEI Quanta FEG 250) at 20 kV and 10000× magnification. The distribution of fiber diameter was determined by analyzing 100 random fibers from SEM image. The produced nanofiber films were stored at 4 °C in a closed container.

### 4.5 Antifungal Activity of Nanofiber Films

The experiment of *in vitro* antifungal activity of nanofiber films was performed by the method adapted from Shi et al.^95^ First, 10 mg of each nanofiber film (CEO, CLEO, and PVA) was weighed and placed into 1.5 mL tubes as duplicates. The 1 mL of spore suspension (peptone water including 1 g/1 L Tween 80) containing 10^3^ spores/mL was added into the tubes. The tube, including PVA was selected as negative control against EO nanofiber films. They were incubated for 24 h at 25 °C. Then, the spread plate method with DRBC agar was performed, and plates were left 2 days of incubation at 25 °C to count fungal colonies.

To evaluate the effectiveness of CEO and CLEO nanofiber films as antimicrobial food packaging, they were applied to organic bread samples. The collected bread samples were sliced into small pieces and placed into Petri dishes as packed inside cling wrap to inhibit moisture loss either with or without nanofiber films (CEO, CLEO, and PVA) and as nonpackaged. They were stored at around 25 °C until the sixth day under sterile conditions.

### 4.6 Statistical Analysis

All the experiments were performed at least in triplicate. Excel 2016 and SPSS software (Version 16.0) were used to analyze the resulting data. One-way analysis of variance (ANOVA), Tukey’s range test, and Duncan’s new multiple range test were applied at 0.05 significance level. Results were reported as mean value ± standard deviations.

## Supporting Information Available

Additional experimental details; disc diffusion assay plates for antifungal activity of EOs against fungal cultures (PDF)
