# Supplementary material for: Antifungal Activities of Different Essential Oils and Their Electrospun Nanofibers against Aspergillus and Penicillium Species Isolated from Bread
Source: ACS Omega. 2022 Oct 14;7(42):37943–53. doi: 10.1021/acsomega.2c05105 (PMC9609062; doi:10.1021/acsomega.2c05105)
Supplement: Supplementary file 1 — ao2c05105_si_001.pdf [file ao2c05105_si_001.pdf]

Supporting Information for Review Only

Antifungal activities of different essential oils and their electrospun nanofibers against *Aspergillus* and *Penicillium* species isolated from bread

Dilara Devecioglu[1], Mustafa Turker[2], Funda Karbancioglu-Guler[1*]

[1]Istanbul Technical University, Faculty of Chemical and Metallurgical Engineering, Department of Food Engineering, 34449, Maslak/ İstanbul/Türkiye

[2]Pak Group, R & D Center, Köseköy Mahallesi, Ankara Cad. No. 277, 41310 Kartepe/Kocaeli/Türkiye

*Corresponding Author, e-mail: karbanci@itu.edu.tr

Figure S1. Antifungal activity of essential oils (EOs) against *Penicillium carneum* DDS4*, Aspergillus flavus* DDS6 and *Aspergillus niger* DDS7.

**EOs**	**Day**	***Penicillium carneum***		***Aspergillus flavus***		***Aspergillus niger***	
		**Diffusion**	**Vapour**	**Diffusion**	**Vapour**	**Diffusion**	**Vapour**
**BPEO**	**3rd day**	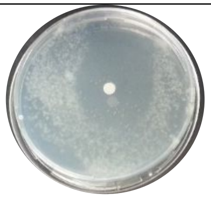	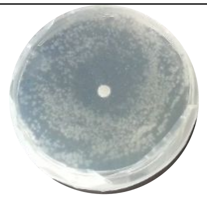	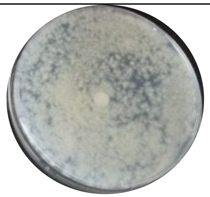	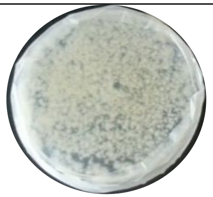	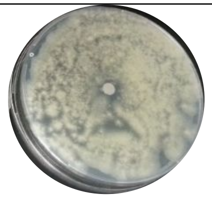	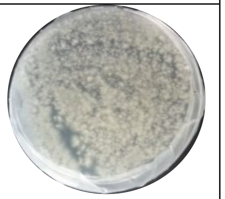
	**6th day**	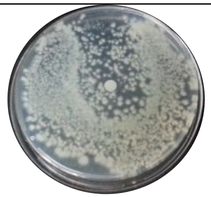	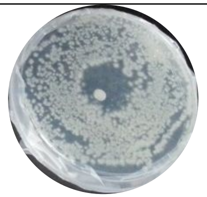	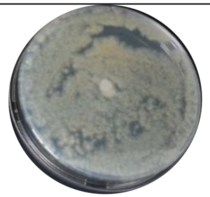	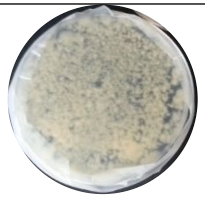	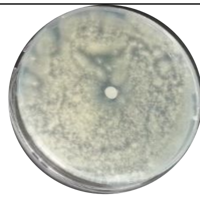	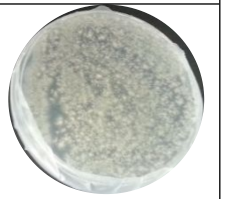
**CEO**	**3rd day**	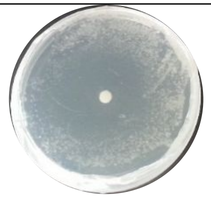	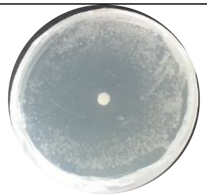	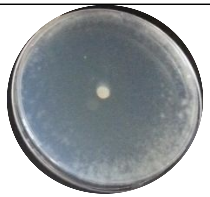	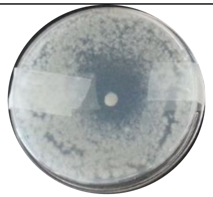	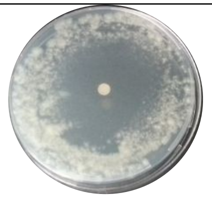	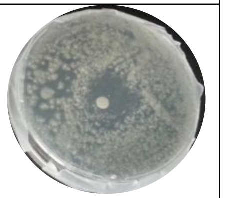
	**6th day**	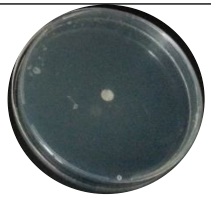	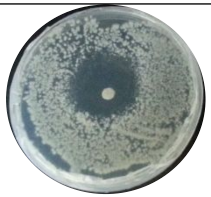	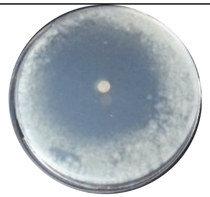	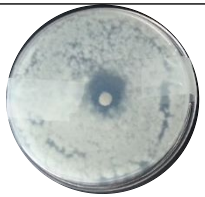	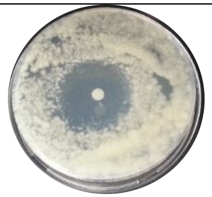	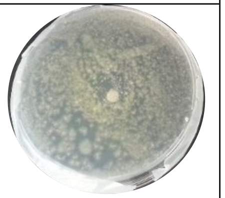

CLEO	**3rd day**	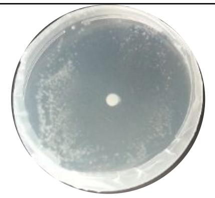	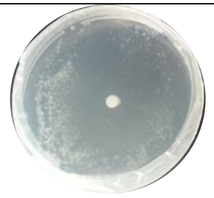	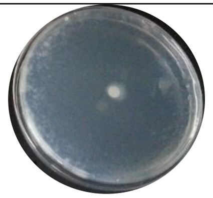	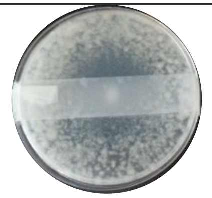	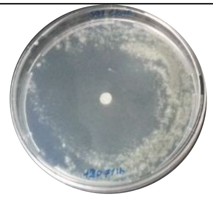	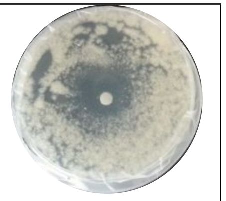
	**6th day**	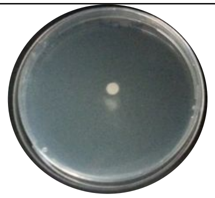	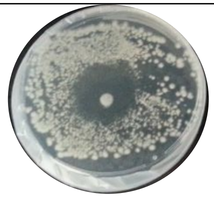	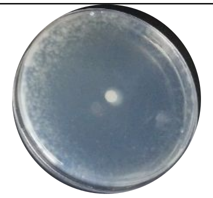	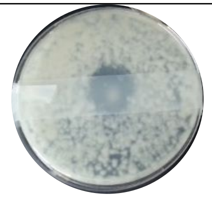	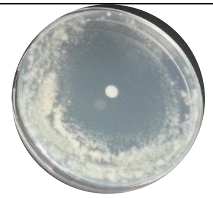	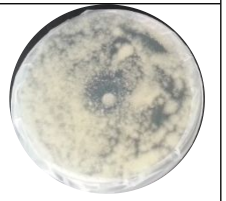
CUEO	**3rd day**	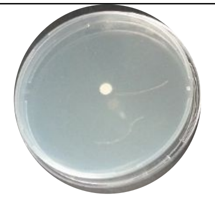	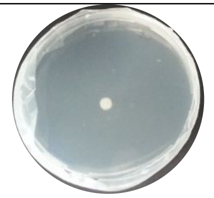	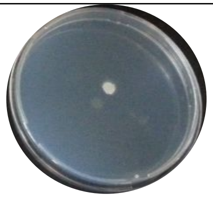	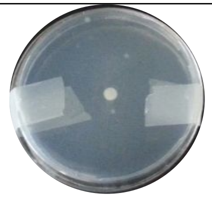	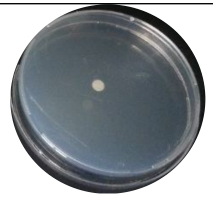	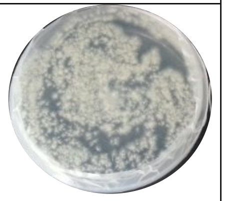
	**6th day**	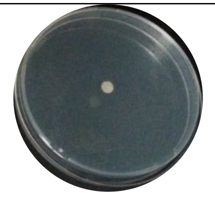	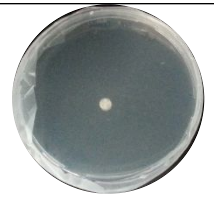	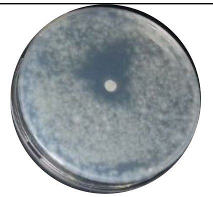	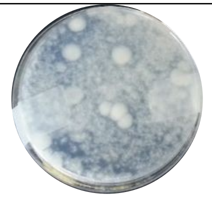	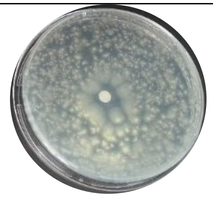	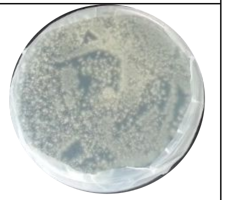
GEO	**3rd day**	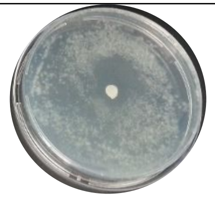	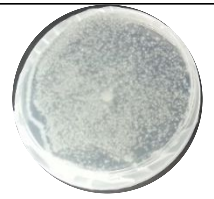	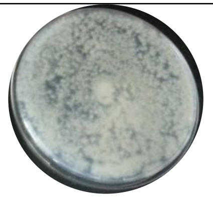	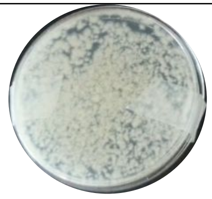	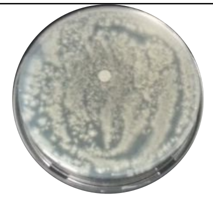	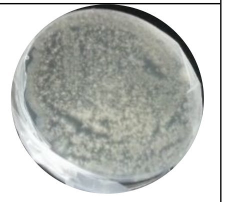

	**6th day**	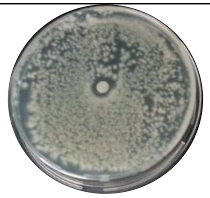	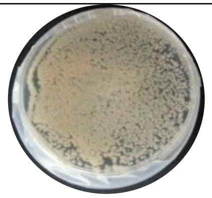	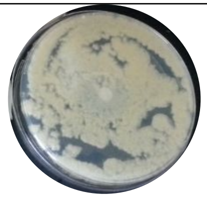	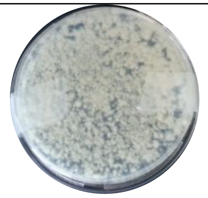	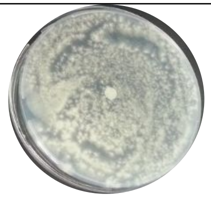	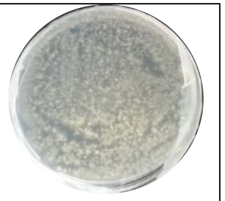
**OEO**	**3rd day**	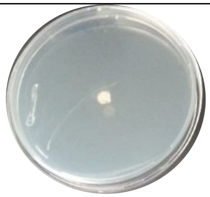	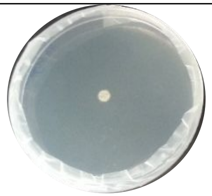	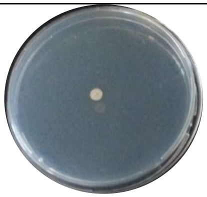	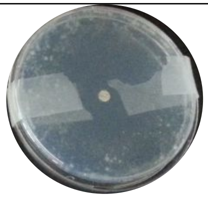	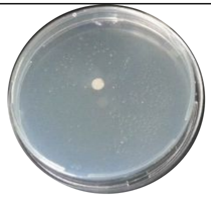	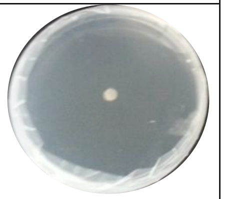
	**6th day**	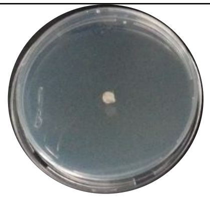	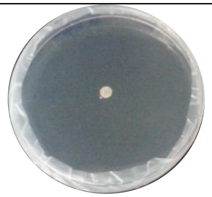	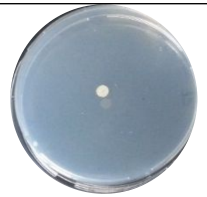	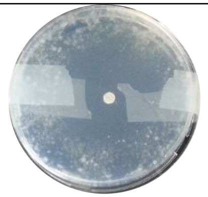	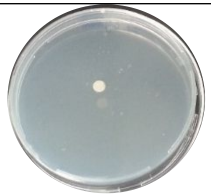	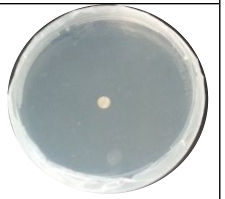

BPEO: black pepper EO, CEO: cinnamon EO, CLEO: clove EO, CUEO: cumin EO, GEO: ginger EO, OEO: origanum EO.
